# Perceptions of Factors Associated With Sustainability of Evidence‐Based Nursing Practice: A Sequential Mixed Methods Study

**DOI:** 10.1155/jonm/6680206

**Published:** 2026-05-15

**Authors:** Jie Lai, Yujie Zhang, Jing Chi, Jingxia Miao, Hua Lin, Weixiang Luo, Jiaqi Fu, Shisi Deng, Zihan Guo, Chuhan Zhong, Jianyao Tang, Bingqian Guo, Tingting Yang, Yanni Wu

**Affiliations:** ^1^ Nanfang Hospital, Southern Medical University, Guangzhou, China, fimmu.com; ^2^ School of Nursing, Southern Medical University, Guangzhou, China, fimmu.com; ^3^ The Second Affiliated Hospital School of Medicine, South China University of Technology, Guangzhou, China, scut.edu.cn; ^4^ The Second Nanning People’s Hospital, Nanning, China; ^5^ Shenzhen People’s Hospital (The First Affiliated Hospital, Southern University of Science and Technology, The Second Clinical Medical College, Jinan University), Shenzhen, China, jnu.edu.cn

**Keywords:** barrier, CFIR, evidence-based nursing practice, facilitator, mixed-methods, sustainability

## Abstract

**Background:**

To improve the quality of nursing services and patient outcomes, the implementation of evidence‐based nursing practice (EBNP) becomes increasingly important. Little is known about determinants to sustainability of EBNP.

**Objective:**

To explore potential barriers and facilitators in the sustainability of EBNP from Chinese nurses’ perspectives.

**Methods:**

A cross‐sectional survey and semistructured interviews were conducted among Chinese nurses participating in evidence‐based practice (EBP) between May to June 2023 and June to July 2024. The interviews and answers to the open‐ended questions were analyzed using content analysis. Based on the length of time that EBNP has been routinely applied in clinical settings, we divided the time into seven stages from not routinely used to greater than 5 years. Descriptive statistics evaluated barriers and facilitators influencing sustainability in different stages.

**Results:**

Of 406 nurses surveyed, 319 provided valid responses. Fifteen nurses participated in interviews. Among 48 constructs in updated Consolidated Framework for Implementation Research, participants reported 3 as barriers, 7 as facilitators, and 21 as both barriers and facilitators. The remaining 17 constructs were not mentioned by participants or were mentioned with insufficient frequency to be categorized as either barriers or facilitators. Major facilitators included benefits to patients and professionals, access to information technology infrastructure, commitment, competent leadership, team and leadership support, incentives, training, assessment of patients’ and professionals’ needs, regular tailoring of strategies, and quality control measures/supervision. Barriers across the sustainment phases included heavy workload, staffing shortages, limited leadership support, low compliance, and low enthusiasm among nurses, even after EBP had been sustained for over 5 years.

**Conclusion:**

The findings enhance understanding of factors promoting the sustainability of EBP from nurses’ perspectives. They highlight that dynamically identifying related barriers provides lessons for designing strategies and programs that promote the sustainability of EBNP in China.

## 1. Introduction

Evidence‐based nursing practice (EBNP) is internationally recognized as a way to rapidly introduce evidence into clinical nursing, improve the quality of nursing services, and achieve better patient outcomes. To date, EBNP has flourished worldwide, with an increasing number of practitioners integrating the evidence into clinical nursing and expecting to improve patient outcomes. Quality improvement (QI) initiatives often build on EBNP by using evidence to design and sustain interventions that enhance healthcare quality and patient outcomes. QI provides a structured approach to implement EBNP, ensuring that evidence‐based interventions are maintained over time in clinical settings. However, many EBNPs are difficult to sustain in the long term after implementation. Research suggests that most evidence fades over time and does not persist for a year [1–3]. Evidence that cannot be sustained over time not only leads to wasted upfront investment but also undermines practitioners’ confidence in undertaking EBNP [4]. Sustainment refers to the ongoing formulation of processes, practices, or work routines that are delivered and learned through the intervention [2].

Over the past decade, much international evidence has highlighted barriers to evidence‐based practice (EBP), including nurses’ lack of EBNP knowledge and skills and organizations’ lack of support for EBNP [5–7]. However, these studies focused on identifying influencing factors during the implementation phase of EBNP and lacked postimplementation studies investigating factors that influence sustainability in healthcare on a global scale. This may be due to EBNP practitioners focusing primarily on the initial uptake and implementation of healthcare innovation in the clinical setting, with less attention paid to the postimplementation challenges of scaling up and sustaining interventions supported by evidence [2]. However, the implementation of EBNP does not guarantee its long‐term sustainability in clinical practice [8]. Moreover, a longitudinal study by Rogal et al. found that the factors influencing the sustainability of EBPs change over time [9]. Therefore, there is still a need to explore the determinants of the postimplementation phase of EBNP to tailor implementation strategies to promote the sustainability of the evidence. Sustainability refers to EBNP delivering its intended benefits over an extended period of time after implementation support is terminated [10]. In this study, we primarily use the term sustainability to refer to the long‐term maintenance and intended benefit of EBNP in clinical settings. The term sustainment is occasionally used to refer specifically to the postimplementation phase during which practices are integrated into routine care.

Understanding the determinants of EBNP sustainability is essential for developing targeted strategies that support the enduring application of evidence in clinical nursing. By identifying these factors, healthcare institutions can optimize resource allocation, enhance nursing quality, and ultimately improve patient outcomes through sustained evidence‐based care. This study aims to explore the barriers and facilitators influencing the sustainability of EBNP in clinical nursing settings.

Several studies have investigated the sustainability of EBP in mental healthcare, prevention, schools, primary care, and acute care settings [2]. However, these studies focus on specific evidence topics and lack a comprehensive description of the determinants that influence long‐term sustainability. In 2022, a study outlining the determinants influencing school‐based public health interventions found that the main barriers were practice and resource constraints and the main facilitators were senior leadership support and staff attentiveness to the positive impact of interventions [11]. However, this study explored the determinants of the sustainability of school‐based interventions rather than those in clinical nursing settings. To our knowledge, implementing evidence in clinical nursing settings may face more complex determinants than implementing evidence in schools. As such, there remains a need to describe the barriers and facilitators to the long‐term sustainability of EBNP in clinical nursing settings.

To date, reviews have noted that common facilitators include clear roles and responsibilities for accountability, while a common barrier has been inadequate staff resources [12]. Moreover, newer reviews highlight persistent conceptual and methodological gaps in the sustainability literature, such as inconsistent definitions and a shortage of long‐term evaluations, which limit our ability to draw actionable conclusions across settings and health systems [13]. However, much of the existing evidence has been generated in high‐income contexts and discussed at the organizational or hospital level, which may not fully capture sustainability factors in frontline clinical nursing units (e.g., inpatient wards, outpatient clinics, and ICUs) where nurses operationalize evidence through day‐to‐day care processes.

EBNP has expanded rapidly through professional initiatives and hospital‐led implementation projects in China [14]. Nevertheless, unlike some high‐income settings where EBP is mandated through regulatory authorities, EBNP implementation is not a standardized requirement for all registered nurses [6]. The Chinese Nursing Association promotes EBNP as part of professional development. However, adoption primarily depends on hospital initiatives and leadership directives [5], which explains the varying stages of EBNP implementation observed in our study, as healthcare institutions are at different points in their EBP journey. A national scoping review of EBP implementation research in China documented a sharp increase in publications, predominantly from nursing; however, the existing literature focuses largely on implementation activities and short‐term outcomes rather than sustainability outcomes [15].

These suggest that there is a lack of focus on how EBNP is sustained by EBNP practitioners in China, which will make it difficult for EBNP to continue to be sustained in the clinical setting beyond the implementation phase. The more EBNPs are implemented, the more money and resources nurse practitioners invest in improving nursing quality. In addition, a study has found that implementing evidence in low‐ and middle‐income countries requires greater economic investment than in developed countries [16]. As such, if China’s EBNPs were to fade over time, then EBNP practitioners would have wasted their enormous efforts and would not be able to capture the long‐term benefits of EBNPs. It is, therefore, imperative to explore the barriers and facilitators affecting the sustainability of EBNP in China to avoid wasted up‐front investment and reap long‐term benefits. The purpose of this study was to explore the barriers and facilitators that influence the sustainability of EBNP. We developed this research question based on the differences observed in real‐world practice: Why are certain EBP programs unable to be sustained in the Chinese context? What are the barriers and facilitators?

## 2. Methods

### 2.1. Design

This study employed an explanatory sequential mixed‐methods design, including an online cross‐sectional survey and semistructured interviews. First, we aim to identify the full scope of barriers and facilitators to sustaining EBNP through a survey. These results served as the basis for the qualitative exploration of barriers and facilitators in the next phase. Second, semistructured interviews were conducted to explain further and clarify the results and reasons. The reporting adheres to the Consolidated Criteria for Reporting Qualitative Research (COREQ) [17] and Good Reporting of A Mixed Methods Study (GRAMMS) guidelines [18] (Supporting files 1 and 2).

### 2.2. Participants and Sampling

#### 2.2.1. The Quantitative Phase

For the quantitative component, convenience and snowball sampling were used. Selection criteria included (1) being a registered nurse in China; (2) having conducted or participated in at least one EBNP project; and (3) willingness to share experiences regarding barriers and facilitators to EBNP sustainability. A poster with a QR code for the survey was sent to a WeChat group containing 360 nurses from 121 hospitals for EBNP. We invited nurses in the group who had conducted or participated in an EBNP in China to complete the survey and share the poster with others. WeChat is one of the most widely used social media platforms in China, and the Nanfang Nursing Center for Evidence‐Based Practice formed a WeChat group. The Nanfang Nursing Centre is a Joanna Briggs Institute (JBI) Centre of Excellence based in Guangzhou, Guangdong Province, China. Established in 2017, the Nanfang Nursing Centre’s primary mission is to support and promote the use of the best available evidence for hospitals to improve care delivery. It has successfully guided 74 hospitals in 32 cities in China to implement EBNP in 2022 [19].

#### 2.2.2. The Qualitative Phase

For the qualitative component, purposive sampling was employed to recruit participants with rich information about the phenomenon under study. During a debriefing session for the EBNP project in Guangdong province, project leaders from across China who had already conducted an EBNP program and had fully responded to our invitation to participate in semistructured, face‐to‐face interviews were invited to participate. Selection criteria for interview participants included (1) being a project leader of at least one EBNP initiative; (2) having comprehensive knowledge of the entire implementation process; and (3) representing EBNP projects with varying durations of implementation to ensure diverse perspectives. We aimed to include healthcare nurses with EBNP experience, ranging from projects that failed to sustain to those sustained for more than 2 years. Project leaders were chosen because they were involved in the entire process and were most likely to have an in‐depth understanding of barriers and facilitators in the sustainability of EBNP. Finally, 15 participants were included in the final sample: 13 were interviewed face‐to‐face and 2 were interviewed by telephone due to their work schedules.

### 2.3. Data Collection

#### 2.3.1. Survey Development and Data Collection

The survey consisted of two parts, one of which was the demographic and professional characteristics of the participants, including gender, age, education, profession, level of qualification, job title, work position, work experience, level of the hospital, province where the hospital is located, whether or not an EBNP project has been carried out, the topic of the EBNP project, the research design, theories, models, frameworks used for the EBNP, whether or not the EBNP is currently being used in the clinical setting on a routine manner, and the length of time that the EBNP has been used in the clinical setting at this point.

In the second part, two open‐ended questions were used, and respondents were asked to provide written responses. The first open‐ended question was “What do you think are the most significant influences that impede the sustainability of EBNP programs in the clinical setting? Tell us your story.” The second open‐ended question was “What do you think are the most significant influences that facilitate the sustainability of EBNP programs in the clinical setting? Tell us your story.”

The survey, released on the online survey platform Questionstar (https://www.wjx.cn), began with an informed consent statement, which participants agreed to by completing the survey. Nurses can scan the QR code on the poster to fill out an anonymous online survey. The information was collected and stored in the password‐protected survey Star platform, and only designated members of the researcher’s team had access to the raw data. Survey data collection was conducted between May and June 2023.

#### 2.3.2. Qualitative Data Collection

Quantitative findings showed limited sustaining of EBNP and brief descriptions in the open‐ended responses. To explore and interpret factors influencing the sustainability of EBNP, an interview guide was created based on the results of the quantitative phases, using the five domains of the Consolidated Framework for Implementation Research (CFIR), including innovation, outer setting, inner setting, individuals, and process [20]. The interview guide was developed collaboratively after a group discussion with the study authors. The interview guide was presented in Supporting file 3. Before the interviews, we invited two nurses who have conducted EBNP in our hospital to preinterview and demonstrate their capacity to explore barriers and facilitators affecting EBNP sustainability using the interview guide. All interviews were conducted by two trained qualitative researchers (Jie Lai and Yujie Zhang).

Demographic characteristics were collected manually before the interview started. Each participant was interviewed in Chinese. The length of interviews ranges from 22 to 45 min (mean 30 min). All interviews were audio recorded and transcribed verbatim. Interviews continued until data saturation was reached, as no new information emerged. After conducting and analyzing 13 interviews, we observed that no substantially new themes were emerging. We conducted two additional interviews to confirm saturation, bringing the total to 15 interviews. These final interviews yielded no new themes or significant variations to existing themes, confirming that data saturation had been reached. All interviews were conducted between June and July 2024.

### 2.4. Data Analysis

#### 2.4.1. Quantitative Data Analysis

We organized and described quantitative survey data as frequency counts and proportions of the total sample. Basic information on participants was analyzed using descriptive statistics and presented as counts (%). Pearson’s chi‐square test was used to examine the association between TMF use and the routine application of EBNP in clinical practice, with Fisher’s exact test applied when the expected cell counts were less than 5 [21]. Statistical significance was set at *p* < 0.05. As we collected the length of time that EBNP has been routinely applied to the clinical, we divided the time into seven stages, as follows: (1) not routinely used (S1), (2) less than or equal to 1 year (S2), (3) greater than 1 and less than or equal to 2 years (S3), (4) greater than 2 and less than or equal to 3 years (S4), (5) greater than 3 and less than or equal to 4 years (S5), (6) greater than 4 and less than or equal to 5 years (S6), and (7) greater than 5 years (S7). The frequency of each subtheme of barriers and facilitators that appeared in each of the seven stages was summarized descriptively. IBM SPSS Statistics for Windows (Version 26.0, IBM Corp., Armonk, NY, USA) was used for data analysis.

#### 2.4.2. Qualitative Data Analysis

Directed content analysis, guided by the CFIR 2.0, was used to analyze qualitative data obtained from interviews and two free‐text responses. This method is a structured qualitative analysis approach that employs an existing theoretical framework to direct the coding process while allowing for the identification of new themes. The qualitative data were imported into NVivo (Version 14.0, QSR International, Melbourne, Australia). Study members included a Ph.D. in nursing and two graduate nursing students. All members had experience with narrative data analysis and had implemented or mentored EBNP. After reading the texts for immersion to familiarize themselves with the texts, two researchers (Jie Lai and Yujie Zhang) independently coded the data and generated subthemes of barriers and facilitators using the CFIR structures. After completing the coding, the two fellows mentioned above exchanged their codes and worked together to identify any discrepancies. Any inconsistencies are discussed and categorized by the two fellows. If the two fellows could not reach a consensus, a third fellow would be invited to have a group discussion, and the third fellow (Yanni Wu) would decide how to code and categorize.

CFIR is one of the most frequently cited theories in implementation science, first published in 2009 and updated in 2022.

CFIR is often used to predict or explain barriers and facilitators to effective implementation or to retrospectively explain implementation outcomes by evaluating the determinants in different implementation contexts. The updated CFIR contains 5 domains, 48 structures, and 19 substructures, of which the five domains are the innovation, the outer setting, the inner setting, the individuals (role subdomain and characteristics subdomain), and the implementation process domain [20]. All responses from valid surveys and interviews were included in the analysis, except those that were referenced only briefly due to insufficient context for meaningful categorization.

#### 2.4.3. Mixed‐Method Integration

To synthesize these data, a joint display approach was utilized to align quantitative results with emerging qualitative themes [22, 23]. All authors participated in discussions throughout integration process.

### 2.5. Sample Size Calculation for Survey and Interviews

We followed the guideline by Tran [24], which estimated that the point of sample adequacy was reached for samples > 150 participants in a survey with open‐ended questions. Accounting for approximately 20% of invalid or incomplete surveys, the target sample size was calculated to be 188. During the data collection process, we adopted a strategy of collecting and analyzing data simultaneously until sample adequacy for diverse responses was reached [25]. Given the need to collect structured survey items and open‐ended questions, we used this estimation to collect more prevalent and salient ideas from both quantitative summaries and qualitative insights. For the qualitative portion, we planned to recruit 15 to 20 participants and conduct interviews until data saturation is achieved, determined when no new information emerges, indicating that the sample size is sufficient [26].

### 2.6. Reflexivity, Rigor, Validity, and Trustworthiness

For quantitative part, the survey was developed by a Doctor of Philosophy (PhD) and postgraduate supervisor in nursing and by six postgraduate nursing students following a group discussion. They all have experience in conducting EBNP, and one of them is a JBI‐accredited Evidence‐Based Training Instructor. To ensure data quality, the survey platform restricted duplicate submissions for the same participant and required completion of key items to minimize missing data. As we adopted a strategy of collecting and analyzing data simultaneously, data were screened for validity and completeness. Surveys were excluded if the open‐ended responses contained no substantive content (e.g., None or I do not know) or were, otherwise, not interpretable for analysis.

For qualitative part, a systematic process ensured accurate translation of Chinese‐language data. Interviews were conducted in Chinese, transcribed verbatim, and translated into English by a bilingual researcher with a UK PhD in nursing. A second bilingual researcher, experienced in English‐language qualitative studies, verified accuracy through backtranslation, with discrepancies resolved via team discussion. Study materials (e.g., interview guides and surveys) were developed in Chinese and translated into English by bilingual team members, preserving cultural fidelity. Methods and qualitative findings, analyzed in Chinese, were translated into English postanalysis, maintaining contextual meaning. Representative quotes underwent rigorous translation and backtranslation. Rigor was upheld by qualified bilingual researchers, backtranslation, and team consensus, ensuring accurate and culturally sensitive reporting.

All the researchers adhered to dialectical pragmatism [27] in the use of qualitative and quantitative research methods to collect and analyze data, consolidate findings, and naturally draw inferences from the data. The two interviewers, one interviewer (Female, postgraduate student, registered nurse) and the other interviewer (Male, postgraduate student, registered nurse), did not have established relationships with the interviewees before this study commenced. In addition, another member of the research team (PhD, researcher, registered nurse) is certified as a JBI evidence implementation program trainer. All the researchers are experienced in conducting qualitative interviews and content analysis, have systematically studied and researched EBNP, and have led or conducted EBNP. To ensure the reliability and trustworthiness of the data, the research team maintained a detailed record of data collection and analysis procedures. Multiple researchers reviewed and interpreted the findings through team discussions to enhance consistency and credibility. Nurses who were not part of the study team were consulted to ensure that the survey and interview guides were relevant to clinical practice.

### 2.7. Ethical Considerations

All nurses participating in the interviews provided written consent. Submission of the survey implied that participants had read the informed consent statement and agreed to the anonymized use of their responses for data analysis. The survey was anonymous and completed in a one‐time submission, and once responses were submitted, participants were unable to withdraw their responses. Ethical approval for this study was obtained from the Medical Ethics Committee of Nanfang Hospital, Southern Medical University, Guangzhou, China (Ethical number: NFEC‐2021‐439).

## 3. Results

### 3.1. Characteristics of Participants

A total of 406 nurses completed the survey, of which 87 did not respond effectively to the open‐ended questions, and 319 surveys were finally included, with a validity rate of 78.57%. The 319 nurses came from 19 cities in China, of which 311 nurses worked in tertiary hospitals, seven in secondary hospitals, and one in a community hospital. Of the 319 nurses, 232 nurses indicated that the EBNP for which they were responsible or involved was routinely used in the clinical setting, with approximately half of these nurses indicating that the EBNP had been used in the clinical setting for less than or equal to 1 year and 87 nurses indicated that the EBNP was not routinely used in the clinical setting. In the interview, 17 nurses who are project leaders of EBP projects were purposely invited. Except for 2 nurses’ absences due to work constraints, 15 nurses of evidence‐based nurse practice project responders participated in the interviews. More details are shown in Table 1.

**TABLE 1:** Characteristics of nurses participating in the interviews and survey.

Items	Survey (*n* = 319, %)	Interview (*n* = 15, %)
Gender		
Male	12 (3.76)	1 (6.67)
Female	307 (96.24)	14 (93.33)
Age		
19–30	74 (23.2)	7 (46.67)
31–60	244 (76.49)	8 (53.33)
> 60	1 (0.31)	0 (0)
Education background		
Associate degree	1 (0.31)	0 (0)
Bachelor’s degree	145 (45.46)	2 (13.33)
Master’s degree	167 (52.35)	11 (73.34)
Doctors’ degrees	6 (1.88)	2 (13.33)
Roles		
Nurse	217 (68.02)	11 (73.34)
Head nurse	85 (26.65)	2 (13.33)
Vice Director of Nursing	2 (0.63)	1 (6.67)
Director of Nursing	15 (4.70)	1 (6.67)
Job title		
Registered Nurse	14 (4.39)	0 (0)
Nurse Practitioner	53 (16.61)	5 (33.33)
Supervising Nurse Practitioner	168 (52.66)	8 (53.33)
Associate Chief Nurse Practitioner	56 (17.55)	2 (13.33)
Chief Nurse Practitioner	28 (8.79)	0 (0)
Years of working experience		
0–5	65 (20.38)	5 (33.33)
6–10	73 (22.88)	5 (33.33)
11–15	103 (32.29)	5 (33.33)
16–20	36 (11.29)	0 (0)
> 20	42 (13.16)	0 (0)

### 3.2. The Relationship Between Having Theories, Models, and Frameworks to Guide and the Ability of EBNP to Be Routinely Applied in Clinical Settings

EBNP, guided by Theories, Models, and Frameworks (TMFs), was more routinely used and sustained in clinical settings than without a TMF, with a ratio of 78.82% versus 62.07%, *p* < 0.001 (Table 2).

**TABLE 2:** The relationship between having theories, models, and frameworks (TMFs) to guide and the ability to be routinely applied in clinical settings (*n* = 319).

Group	Whether evidence‐based nursing practice is routinely applied in clinical		Total
	No	Yes	
No TMFs to guide	44 (37.93%)	72 (62.07%)	116
Guided by TMFs	43 (21.18%)	160 (78.82%)	203
Total	87	232	319
*χ* ^2^	10.440		
*p*	< 0.001		

Abbreviation: TMFs, Theories, Models, and Frameworks.

### 3.3. Barriers and Facilitators Affecting EBNP

**FIGURE 1:**
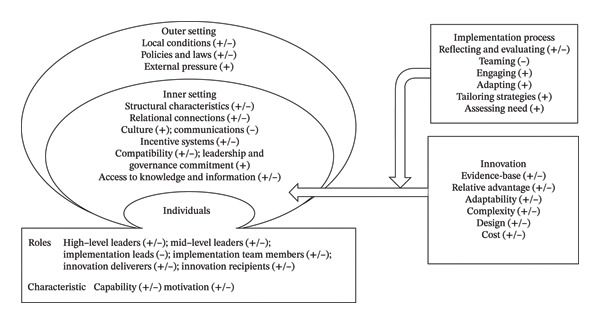
Identified barriers and facilitators influencing the sustainability of EBNP based on CFIR 2.0 constructs. Note: +, facilitators; −, barriers. EBNP, evidence‐based nursing practice; CFIR, Consolidated Framework for Implementation Research.

A frequency of 394 barriers and 377 facilitators was mentioned in 319 nurses’ responses. We identified 30 factors from the CFIR 2.0 constructs that influence the sustainability of EBNP, grouped into five domains: innovation, the external environment, the organizational context, individual factors, and the implementation process (Figure 1). The themes and subthemes identified from the open‐ended surveys, along with their frequencies across the seven phases, are described in Tables 3 and 4. The survey and interview responses are used to clarify and illustrate the relevant factors throughout the Results section. Each interview participant was continuously numbered as Participant 1, 2, etc. A joint display was provided to further describe the integration of the results (Supporting file 4).

**TABLE 3:** Barriers of the sustainability of evidence‐based nursing practice.

Domain of CFIR	Subtheme of barrier	Not routinely used in the clinic (S1)	Years of routine clinical use						Number of stages mentioned out of 7 stages (*n* = 7)	Total
			≤ 1 (S2)	> 1‐≤ 2 (S2)	> 2‐≤ 3 (S3)	> 3‐≤ 4 (S5)	> 4‐≤ 5 (S6)	> 5 (S7)		
I. Innovation domain										
Construct name										
Innovation evidence‐based	Lack of evidence	1 (0.92)	3 (2.13)		2 (5.26)			1 (7.69)	4	7 (1.78)

Innovation relative advantage	Lack of practicality	1 (0.92)	1 (0.71)	1 (1.18)		1 (20)			4	4 (1.02)
	Failure to deliver economic benefits	3 (2.75)							1	3 (0.76)
	Lack of significant change									
	Failure to improve patient outcomes		1 (0.71)						1	1 (0.25)

Innovation adaptability	Evidence not adapted to a local clinical context	2 (1.83)	3 (2.13)	3 (3.53)					3	8 (2.03)

Innovation complexity	Complexity of innovation	1 (0.92)	5 (3.55)	2 (2.35)		1 (20)			4	9 (2.28)

Innovation design	Failure to develop a standardized process for innovation		1 (0.71)	3 (3.53)					2	4 (1.02)

Innovation cost	Increased cost of work	**8 (7.34)**	**10 (7.09)**	**7 (8.24)**	**2 (5.26)**	**1 (20)**			**5**	**28 (7.11)**
	Increased operational costs	1 (0.92)		2 (2.35)	1 (2.63)				3	4 (1.02)
	Increased cost of patient care			1 (1.18)	1 (2.63)				2	2 (0.51)

II. Outer setting domain										
Local conditions	Lack of hospital support	1 (0.92)		1 (1.18)	1 (2.63)				3	3 (0.76)

Policies and laws	Not in line with “rules”	1 (0.92)	2 (1.42)						2	3 (0.76)
	Not a chargeable item for hospitals	1 (0.92)	1 (0.71)						2	2 (0.51)

III. Inner setting domain										
Physical infrastructure	Lack of infrastructure		1 (0.71)		1 (2.63)				2	2 (0.51)

Information technology infrastructure	Lack of electronic information system support	2 (1.83)		2 (2.35)	1 (2.63)				3	5 (1.27)

Work infrastructure	High work pressure	**14 (12.84)**	**26 (18.44)**	**11 (12.94)**	**2 (5.26)**			**2 (15.38)**	**5**	**55 (13.96)**
	Insufficient clinical nurse	**13 (11.93)**	**15 (10.64)**	**6 (7.06)**	**5 (13.16)**			**2 (15.38)**	**5**	**41 (10.41)**
	Insufficient research nurses	3 (2.75)	2 (1.42)		2 (5.26)				3	7 (1.78)
	Staff changes	1 (0.92)			1 (2.63)				2	2 (0.51)

Relational connections	Lack of departmental team support	**7 (6.42)**	**10 (7.09)**	**5 (5.88)**	**6 (15.79)**				**4**	**28 (7.11)**

Communications	Inadequate healthcare communication	1 (0.92)							1	1 (0.25)

Compatibility	Noncompliance with existing workflow	2 (1.83)		2 (2.35)					2	4 (1.02)

Incentive systems	Lack of incentives		1 (0.71)						1	1 (0.25)

Funding	Lack of funding for EBP implementation	1 (0.92)	1 (0.71)	1 (1.18)					3	3 (0.76)

Materials and equipment	Lack of items to implement EBP		2 (1.42)						1	2 (0.51)

Access to knowledge and information	Lack of training		2 (1.42)					1 (7.69)	2	3 (0.76)

IV. Individuals domain										
Roles subdomain										
High‐level leaders	Lack of leadership support	**11 (10.09)**	**7 (4.96)**	**7 (8.24)**	**3 (7.89)**		**1 (33.33)**	**4 (30.77)**	**6**	**33 (8.38)**

Implementation team members	Lack of support from doctors	6 (5.5)	3 (2.13)		1 (2.63)	2 (40)			4	12 (3.05)

Innovation deliverers	Low compliance of nurses to implement EBNP	**3 (2.75)**	**5 (3.55)**	**5 (5.88)**	**2 (5.26)**		**1 (33.33)**	**1 (7.69)**	**6**	**17 (4.31)**
	Low enthusiasm for nurses to participate in EBNP	**4 (3.67)**	**5 (3.55)**	**2 (2.35)**	**1 (2.63)**			**1 (7.69)**	**5**	**13 (3.3)**
	Lack of nurse ownership		1 (0.71)						1	1 (0.25)
	New nurses need training			1 (1.18)	1 (2.63)				2	2 (0.51)

Innovation recipients	Low patient and family compliance	**1 (0.92)**	**10 (7.09)**	**4 (4.71)**	**1 (2.63)**				**4**	**16 (4.06)**

Characteristics subdomain										
Capability	Lack of knowledge among nurses	**5 (4.59)**	**2 (1.42)**	**3 (3.53)**	**1 (2.63)**				**4**	**11 (2.79)**
	Lack of leadership by those in charge	5 (4.59)	3 (2.13)						2	8 (2.03)
	Nurses lack evidence‐based competence			3 (3.53)					1	3 (0.76)

Motivation	Practitioner inertia	4 (3.67)	10 (7.09)	1 (1.18)				1 (7.69)	4	16 (4.06)
	Disapproval of EBNP by doctors and nurses	**3 (2.75)**	**6 (4.26)**	**8 (9.41)**	**1 (2.63)**		**1 (33.33)**		**5**	**19 (4.82)**

V. Implementation process domain										
Teaming	Difficulty in forming implementation team		1 (0.71)						1	1 (0.25)

Reflecting and evaluating	Lack of quality control measures	**3 (2.75)**	**1 (0.71)**	**4 (4.71)**	**2 (5.26)**				**4**	**10 (2.54)**

*Note:* S1–S7 correspond to different durations of routine clinical use: S1 = not routinely used, S2 = less than or equal to 1 year, S3 = greater than 1 and less than or equal to 2 years, S4 = greater than 2 and less than or equal to 3 years, S5 = greater than 3 and less than or equal to 4 years, S6 = greater than 4 and less than or equal to 5 years, S7 = greater than 5 years. The bold values represent the barriers that were frequently mentioned by nurses across the five domains of CFIR.

Abbreviations: CFIR, Consolidated Framework for Implementation Research; EBNP, evidence‐based nursing practice; EBP, evidence‐based practice.

**TABLE 4:** Facilitators of evidence‐based nursing practice sustainability.

Domain of CFIR	Subtheme of facilitator	Not routinely used in the clinic (S1)	Years of routine clinical use						Number of phases mentioned out of 7 phases(*n* = 7)	Total
			≤ 1 (S2)	> 1‐≤ 2 (S3)	> 2‐≤ 3 (S4)	> 3‐≤ 4 (S5)	> 4‐≤ 5 (S6)	> 5 (S7)		
I. Innovation domain										
Construct name										
Innovation evidence‐based	Supported by scientific evidence	6 (5.61)	4 (3.42)	4 (4.12)					3	14 (3.71)

Innovation relative advantage	Practicality	1 (0.93)	2 (1.71)	1 (1.03)					3	4 (1.06)
	Economic benefits	1 (0.93)							1	1 (0.27)
	Significant clinical effect	4 (3.74)	2 (1.71)	5 (5.15)			1 (33.33)		4	12 (3.18)
	Benefits to patients	**5 (4.67)**	**8 (6.84)**	**7 (7.22)**	**2 (5.71)**	**1 (20)**		**1 (7.69)**	**6**	**24 (6.37)**
	Beneficial to nurses and patients		1 (0.85)						1	1 (0.27)
	Improves work efficiency	**3 (2.8)**	**7 (5.98)**	**5 (5.15)**		**1 (20)**	**1 (33.33)**		**5**	**17 (4.51)**

Innovation adaptability	Adaptable to clinical setting	1 (0.93)	4 (3.42)	2 (2.06)					3	7 (1.86)

Innovation trialability	Can be piloted	1 (0.93)							1	1 (0.27)

Innovation complexity	Simple and easy to implement	2 (1.87)	2 (1.71)	3 (3.09)		1 (20)			4	8 (2.12)

Innovation design	Standardize the innovation	2 (1.87)	1 (0.85)						2	3 (0.8)

Innovation cost	Cost‐neutral or low cost		1 (0.85)	1 (1.03)					2	2 (0.53)

II. Outer setting domain										
Local conditions	Organizational support	**2 (1.87)**	**4 (3.42)**	**3 (3.09)**	**1 (2.86)**				**4**	**10 (2.65)**

Policies and laws	Policy support	**8 (7.48)**	**1 (0.85)**						**2**	**9 (2.39)**

III. Inner setting domain										
Structural characteristics										
Information technology infrastructure	Embedded in information system	**1 (0.93)**	**1 (0.85)**	**1 (1.03)**	**1 (2.86)**			**1 (7.69)**	**5**	**5 (1.33)**

Work infrastructure	Reduced work pressure		2 (1.71)						1	2 (0.53)
	Adequate human resources	2 (1.87)	6 (5.13)	3 (3.09)	1 (2.86)				4	12 (3.18)
	Increase research nurses	4 (3.74)	1 (0.85)						2	5 (1.33)

Relational connections	Cooperation of departmental team	**5 (4.67)**	**8 (6.84)**	**8 (8.25)**	**6 (17.14)**				**4**	**27 (7.16)**

Culture										
Learning‐centeredness	Climate for evidence‐based nursing			1 (1.03)				1 (7.69)	2	2 (0.53)

Tension for change	Urgency to implement EBP	1 (0.93)							1	1 (0.27)

Compatibility	Compliance with workflow	2 (1.87)		2 (2.06)					2	4 (1.06)

Incentive systems	Incentives	**3 (2.8)**	**2 (1.71)**	**1 (1.03)**	**1 (2.86)**			**1 (7.69)**	**5**	**8 (2.12)**

Funding	Funding		1 (0.85)	1 (1.03)	1 (2.86)				3	3 (0.8)

Materials and equipment	Adequate material for implementation		1 (0.85)						1	1 (0.27)

Access to knowledge and information	Enhance training	**3 (2.8)**	**2 (1.71)**	**4 (4.12)**	**1 (2.86)**			**2 (15.38)**	**5**	**12 (3.18)**
	Provide expert guidance		1 (0.85)		2 (5.71)				2	3 (0.8)

IV. Individuals domain										
Roles subdomain										
High‐level leaders	Supported by leadership	**23 (21.5)**	**30 (25.64)**	**23 (23.71)**	**10 (28.57)**		**1 (33.33)**	**5 (38.46)**	**6**	**92 (24.4)**

Implementation leads	Persistence of project implementation leaders	2 (1.87)	1 (0.85)						2	3 (0.8)

Implementation team members	Physician support	2 (1.87)		2 (2.06)		1 (20)			3	5 (1.33)

Innovation deliverers	Increased nurse adherence		4 (3.42)	6 (6.19)	2 (5.71)				3	12 (3.18)
	Increased nurse motivation		1 (0.85)						1	1 (0.27)
	Nurses have the authority to lead	1 (0.93)							1	1 (0.27)

Innovation recipients	Patient and family cooperation and support	1 (0.93)	4 (3.42)	3 (3.09)	2 (5.71)				4	10 (2.65)
	EBP meets patient needs		2 (1.71)						1	2 (0.53)

Characteristics subdomain										
Capability	Increased nurse knowledge		1 (0.85)						1	1 (0.27)
	Leadership of leaders	**13 (12.15)**		**2 (2.06)**	**2 (5.71)**	**1 (20)**		**1 (7.69)**	**5**	**19 (5.04)**
	Increased evidence‐based competence of nurses	1 (0.93)	1 (0.85)	1 (1.03)	1 (2.86)				4	4 (1.06)

Motivation	Increased evidence‐based awareness of implementers	2 (1.87)	1 (0.85)	1 (1.03)					3	4 (1.06)
	Healthcare recognizes EBNP	1 (0.93)	3 (2.56)	2 (2.06)	1 (2.86)				4	7 (1.86)

V. Implementation process domain										
Tailoring strategies	Continuity of implementation strategy		1 (0.85)						1	1 (0.27)

Engaging										
Innovation deliverers	Mobilization of healthcare professionals	1 (0.93)	2 (1.71)						2	3 (0.8)

Innovation recipients	Patient engagement		1 (0.85)						1	1 (0.27)

Reflecting and evaluating	Quality control measures	**3 (2.8)**	**3 (2.56)**	**4 (4.12)**	**1 (2.86)**			**1 (7.69)**	**5**	**12 (3.18)**

Adapting	Need for continuous adaptation of EBNP			1 (1.03)					1	1 (0.27)

*Note:* S1–S7 correspond to different durations of routine clinical use: S1 = not routinely used, S2 = less than or equal to 1 year, S3 = greater than 1 and less than or equal to 2 years, S4 = greater than 2 and less than or equal to 3 years, S5 = greater than 3 and less than or equal to 4 years, S6 = greater than 4 and less than or equal to 5 years, S7 = greater than 5 years. The bold values represent the facilitators that were frequently mentioned by nurses across the five domains of CFIR.

Abbreviations: CFIR, Consolidated Framework for Implementation Research; EBNP, evidence‐based nursing practice; EBP, evidence‐based practice.

#### 3.3.1. Innovation

##### 3.3.1.1. Innovation Cost

In this domain, the increased cost of work (28/394, 7.11%) is a frequently mentioned barrier among 6 of 7 stages of evidence implementation. The answers and interviewees stated that the cost of EBNP includes purchasing or exchanging equipment, increasing patients’ treatment costs, and even the time and energy input of healthcare professionals, which may affect the implementation and maintenance of EBNP.

However, nursing department directors believed that costs need to be divided into two levels. Although the economic cost is indeed high, long‐term cost‐effectiveness and social worth promote the sustainability of project.

*“If you simply use the current cost, the economic cost of conducting this project is relatively high. But more and more patients can have the opportunity to receive professional management and promote long-term follow-up, which generates greater social value.” (Participant 9)*

##### 3.3.1.2. Innovation Relative Advantage

“Benefits to patients” (24/377, 6.37%) and “improves work efficiency” (17/377, 4.51%) are related to the innovation relative advantage construct. In 7 stages, where best practice is applied to the clinic, benefits to patients are mentioned repeatedly in 6 stages and improved work efficiency is stated in 5 stages. Nurses explain that if they are informed and witness the positive effect of the EBNP, professionals are willing to continue to implement it, which is a facilitator and motivator to sustain. Participants also emphasized the need for EBNP interventions that improve nurse productivity and are easy for nurses to use in practice.

*“Promoting the EBNP applied into daily practice, the most important point is the embodiment of the effect. After our efforts, the qualified rate is improved, we identified that children′s condition is recovering quickly, the length of hospitalization is shortened, and the incidence of intracranial hemorrhage is also low.” (Participant 6)*

#### 3.3.2. Inner Settings

##### 3.3.2.1. Structural Characteristics

The survey showed that the shortage of nurse staffing (41/394, 10.41%) and heavy workload (55/394, 13.96%) in work infrastructure are very common barriers influencing the EBNP sustainability in the inner setting domain. Participants reported that staffing and workload challenges persisted as barriers even in departments where EBNP had been routinely used for more than 5 years.

From the interview, most interviewees reported that EBNPs can be missed and ignored due to the heavy daily clinical workload and personnel constraints, making it difficult to engage in EBNP sustainability work. It can be consolidated into two barriers: conflicts between additional jobs and the original workflow integration and staff turnover.

For example, interviewees reported that many clinical nurses had shown resistance to adding extra tasks to their original work, leading to low nurse compliance. They reported that many added assessment sheets and record sheets were still manual assessments, which inevitably increased the workload of nurses.

*“Once it is an evidence-based practice that needs to add paper-based assessment content, it is often not well sustained subsequently. Because it inevitably adds to their workload.” (Participant 15)*

Therefore, the support of information technology infrastructure such as embedded in information systems could be a great factor in promoting EBNP sustainability. Most interviewees reported that embedding EBP into medical record system (MRS) or hospital information system (HIS) facilitated easy implementation and regular reminders, which could free up staff and increase nurses’ acceptability and adherence to EBNP.

*“I think it is best to maintain an evidence-based project that is embedded directly into the HIS system. It can form a daily tool that assists our nurses to conduct assessment and implementation every morning or every shift, which can avoid omissions and improve nurse adherence and completion.” (Participant 7)*

In addition, the situation of department and staff turnover adversely affected the implementation of the evidence and training effectiveness, which could also hamper EBNP sustainability.

*“The opening of new units and realignment of physician and nurse groups can affect ……we conducted an EBNP about chemotherapy-induced nausea and vomiting management, aligned with guidelines, and reached a consensus between nurses and physicians. But a new physician group comes here……” (Participant 3)*

##### 3.3.2.2. Relational Connections

The cooperation and support of department team are essential for resolving challenges and promoting the ongoing implementation. In the opened text, 27 of 337 nurses (7.16%) agreed that the cooperation of departmental team was a facilitator of EBNP. The quotes from the survey illustrated *“The cooperation and assistance of the departmental medical and nursing team is needed. Carrying out EBNP sometimes requires the assistance of a multidisciplinary team, not just nurses working alone, and it takes a concerted effort from doctors and nurses together to routinely apply EBNP in the clinical setting.”* Interviewees also reported that a supportive and trustworthy team created a positive feedback cycle that promoted ongoing implementation and long‐term project continuity. Interviewees mentioned that limited communication with other multidisciplinary teams was a barrier. For example, conflicts between doctors’ concepts of treatment and nursing management, or between different roles and responsibilities, led to communication difficulties and hindered the sustainability of EBNP.

##### 3.3.2.3. Incentive Systems

According to the responses, “incentives” (8/377, 2.12%) is a facilitator that nurses mentioned in 5 stages among the 7 stages of evidence implementation. Some nurses stated that their hospitals offer multiple incentives for staff and that these incentives, along with strong departmental support, are strong motivators for developing and maintaining EBNP. For example, leaders provide financial incentives, praise, and free external study opportunities for individuals and departments who are actively involved in EBP projects, thereby boosting rapid adoption and implementation.

Some participants stated that their hospitals had established a performance appraisal system based on EBNP content for nurses, setting up reward or penalty standards, making the effects of maintaining EBNP pay off. Incentive measures can increase nurses’ participation in EBNP, sustain its continuous implementation, and ensure fidelity.

*“If you succeed in completing it, you will get an extra point, and if you fail to complete it, one point will be deducted. We will calculate the money according to the final workload.” (Participant 8)*

##### 3.3.2.4. Access to Knowledge and Information

Nurses also demonstrated the necessity of training to promote sustainability. Nine nurses illustrated that intensive training remains promoted in projects where EBNP is routinely used for more than 1 year. Some hospitals have incorporated relevant EBNP content into the training regulations for new nurses to improve their competence and promote the sustainability of the program. To promote sustainability, some hospitals provide diverse resources for accessing knowledge, such as online learning platforms and learning groups, to spread and update the latest knowledge.

##### 3.3.2.5. Leadership and Governance Commitment

Interviewees emphasized that changing attitudes and norms and embedding behavior change into routine practice required visible and continuous management commitment. In the early stages, implementation was often initiated and maintained by a small number of key staff or champions. Over time, as hospitals recognized EBNP’s importance and demonstrated commitment through formal documents, accountability mechanisms, and incentives, staff were more likely to view it as a long‐term priority and to reduce the suspicion that “*it’s just a passing fad.”* This helped reduce resistance to additional tasks and improved adherence and fidelity, thereby facilitating sustained, internalized behavior change.

#### 3.3.3. Individual

##### 3.3.3.1. High‐Level and Midlevel Leaders

The sustainability of the EBNP has been influenced strongly by leadership. In the survey, a lack of leadership support (33/394, 8.38%) was identified as a significant barrier to sustaining EBNP, while leadership support (92/377, 24.4%) was a facilitator. The survey reply stated the need for top‐down leadership to promote EBNP to establish implementation and care norms.

*“I feel that the support and attention of nursing administrators are essential to enable the long-term use of EBNP in the clinical setting. If EBNP has the participation and support of leaders, then the opposition to the implementation of EBNP in the clinical setting will be reduced and it will be easier to cultivate new work habits among clinical nurses.”*

Interviewees also highlighted that sustaining the projects’ effectiveness requires support of the hospital’s senior leadership, particularly in allocating resources and funding. “*ENBP is not just the change of one department but already involves multiple departments of the hospital. I also want to reform and maintain these things, but the support of my superiors was not sure.” (Participant 8)*

The capability and leadership of leaders (19/377, 5.04%) can be a facilitator. Participants showed that the leadership of leaders plays an important role in establishing a cultural climate that values change and sustainability.

*“Because it is very easy for nurses to form a rigid, experience-based atmosphere. I’m trying to change this clinical situation so that nurses can become flexible, adaptable nurses who don’t stick to the routine. Once you establish a culture climate that supports change, the EBNP can be sustained.” (Participant 3)*

Inversely, some interviewees expressed that some leaders did not view the meaning of EBNP correctly and thought that EBNP is typically implemented as a research project in a clinical nursing setting. Therefore, when the project is terminated, the leader’s support for EBNP in routine work, including funding, training, and supervision, decreases. The implementation of EBNP is becoming unsustainable.

*“Sometimes when I finish an evidence-based practice project, maybe my leader won’t care too much about it, you know? He wasn′t as motivated and supportive as he started on a project.” (Participant 15)*

##### 3.3.3.2. Capability and Motivation

In the survey, the results showed that low compliance of nurses in implementing EBNP (17/394, 4.31%) and low enthusiasm among nurses to participate in EBNP (13/394, 3.3%) are also associated factors hindering the long‐term maintenance of EBNP. Some interviewees mentioned that they never received compensation for their extra tasks, such as assessments and mission work, leading to low enthusiasm among nurses to implement and sustain EBNP. Meanwhile, the lack of knowledge about EBNP among doctors and nurses has led to a lack of acceptance of EBNP. Some participants outline insufficient recognition of effectiveness and necessity, making the project difficult to sustain. Nurses and doctors are more likely to believe in their practice or follow the original regulations.

*“Due to the influence of academic background and seniority, some nurses are mainly empiric, do not recognize the significance of evidence-based practice, and are resistant to carrying out change, which leads to low enthusiasm and compliance.” (Participant 11)*

In contrast, some head nurses noted that the improvement in patient outcomes reflected nurses’ values and that they gained a sense of achievement. Combined with verbal and material incentives, nurses increased their satisfaction and were motivated to maintain the EBNP effect spontaneously.

##### 3.3.3.3. Opinion Leaders and Implementation Leaders

The interviews indicated that an imbalance in authority between new implementation leaders and experienced opinion leaders could be a barrier to sustainability. Opinion leaders are influential individuals whose attitudes about EBNP informally influence others’ behaviors, while implementation leaders are those formally appointed to manage the EBNP implementation process [28, 29]. When experienced opinion leaders held opposing views, new implementation leaders reported limited position and authority to organize, coordinate, and mobilize resources, making them largely dependent on senior staff for decision‐making and support.

*“If my title again promotion, a little more working years, a little more senior may be a little bit more persuasive.” (Participant 11)*

Participants reported that nurses’ attitudes and actions toward long‐term practice were strongly influenced by senior nurses or by nurses who dared to express themselves. At the beginning of the project, different professionals have different views and understandings of EBP. But if important opinion leaders, such as senior and experienced nurses, are included or persuaded by the development of EBNP, other staff will gradually get involved, and EBNP will become part of their daily routine.

*“At the beginning, even if you are two or three people, you should start first, from your small success, and then let others see that this thing is really meaningful and more valuable, and then slowly the whole team will participate and become a daily routine.” (Participant 9)*

#### 3.3.4. Process

##### 3.3.4.1. Tailoring Strategies

Participants described continuous strategy adjustments as facilitators. Continuity of implementation strategy and planning facilitates EBNP sustainability. Only one nurse in a project lasting less than a year responded to the survey, and the continuous adjustment strategy was a motivating factor. However, nearly half of the nurses mentioned during the interview that the departments are dedicated to continuously improving implementation strategies and adapting them to local contexts to promote the long‐term development of the projects.

The preliminary evaluation metrics in the structured assessment tools used at different implementation stages were constantly refined, and the nursing routine process should be continuously revised. Furthermore, interviewees indicated that barriers changed at different stages and that strategies should also be continually adjusted. “*The focus will be a little different. When implemented, we will first analyze whether there are corresponding resources, systems, and evaluation forms at the system level. The second one is basically that nurses are not knowledgeable enough. Later, there will be other new barriers, like the workflow not being clear or simplified enough. It is still necessary to gradually adjust and refine strategies according to your clinical practice.” (Participant 5)*

##### 3.3.4.2. Assessing Needs

Surprisingly, interviewees identified the assessment of patients’ and professionals’ needs as a critical facilitator of the program’s maintenance. However, this was not mentioned in the survey. The project leaders noted that conducting a needs assessment was crucial to understanding the underlying reasons and considerations that influence professionals’ participation and to identifying strategic intervention points. A theoretical framework was even used to guide formal qualitative interviews, which helped foster participants’ willingness to engage in, implement, and sustain the program. By recognizing these factors, the team could develop targeted strategies to increase participation and address potential barriers to the project’s success. This theme did not surface in the survey, likely due to its structured nature, as surveys are typically designed with preset questions that limit open‐ended exploration. In contrast, interviews allowed participants to express themselves freely, underscoring the importance of a needs assessment. Moving forward, it is recommended to integrate needs assessment as a regular practice during the planning phase and to conduct it periodically. This approach will allow interventions to be adjusted based on the real‐world experiences of patients and professionals, ensuring the project’s long‐term sustainability and relevance in clinical practice.

*“If you don′t dig deep into doctors′ and nurses’ ideas and simply use our daily informal communication, they may say that we are too busy or though they have no problem implementing EBNP, they didn’t when it is formally implemented. Because deep factors are not expressed.” (Participant 3)*

##### 3.3.4.3. Reflecting and Evaluating

“Quality control measures” (12/377, 3.18%) were identified as facilitators from the reflection and evaluation construct, based on survey responses. The head nurse proposed that the daily routine of EBNP be supervised through regular inspection by the department or superior. The measures are described as follows: “*There is a need to establish a monitoring system to continuously monitor and improve the nurses’ implementation of the EBNP requirements to promote the long-term maintenance of EBNP.”* It corresponds with the interview, interviewees described using the superior monitoring or department self‐monitoring as enablers of long‐term maintenance. As a result, this increases the compliance of ENBP and promotes practice becoming routine.

*“After conducting an evidence-based practice project, our department assigned specific staff to monitor every three months and conducted at least four rounds of follow-up supervision to maintain and solidify the best practice.” (Participant 4)*

The annual inspection by the government or the city is also a regular quality control measure that promotes sustainability. Moreover, some interviewees reported that they do not embed sustainability into the strategic design and planning of an EBP project, which might hinder the project’s long‐term sustainability.

*“As we didn’t know if the project could be sustained, there are no more plans for sustainability. We just implemented it step by step.” (Participant 11)*

#### 3.3.5. Outer Settings

##### 3.3.5.1. Policies and Laws

Factors related to the outer setting were less frequently mentioned in the survey. However, during the interview, many nurses mentioned that the national policy of big health is that optimizing the quality of care is crucial to long‐term sustainability. The international or Chinese nursing team attaches great importance to the scientific research atmosphere of evidence‐based medicine and can also play a certain role in encouraging and guiding the sustainability of ENBP for nurses.

*“In terms of national policies whether some policies such as healthy China, or when we are implementing them, they are moving forward. Then our scientific research achievements are in the implementation of national policies, which support patients’ better quality of life.” (Participant 3)*

##### 3.3.5.2. Partnerships and Connections

Communication and cooperation in some universities or primary hospitals, the establishment of evidence‐based nursing cooperation groups, and an EBP base will also promote the sustainable development of EBP.

*“Now we have the support that our hospital established the evidence application base in collaboration with the university school of nursing. With this help, we formed a hospital evidence-based team…, managed and conducted by a nursing doctor. (Participant 14)*

## 4. Discussion

Based on the CFIR, a mixed‐methods study was conducted to explore potential barriers and facilitators in the sustainability of EBNP, incorporating the perspectives of nurses in China. By identifying practitioner responses, we found that EBNP guided by TMFs was more likely to be routinely applied and sustained in clinical settings. These findings are consistent with previous explorations of factors that impede and facilitate EBNP implementation [15, 30]. Therefore, nurse practitioners should use a TMF to guide the implementation of EBNP during the implementation phase and consider strategies to promote long‐term sustainability of EBNP. The integrated quantitative and qualitative findings identified the main barriers to the long‐term sustainability of EBNP, including heavy clinical workload (13.96%), insufficient numbers of clinical nurses (10.41%), and limited access to leadership support (8.38%). Moreover, nurse participants also articulated that being strongly supported by leadership (24.4%), leadership of leaders (5.04%), and benefits to patients (6.37%) are the key to advancing sustainability.

Additionally, the benefits of training (3.18%) and quality control measures (3.18%) are revealed and the EBNP and intervention that improve work efficiency (4.51%) are expected to be sustained. The high frequency of these barriers and facilitators across the seven stages (from EBNP not routinely used to last more than 5 years) indicates some issues to be addressed to help sustain the intervention over time. Barriers are primarily found in the individual domain of CFIR, while facilitators are primarily found in the inner setting domain of CFIR. Perhaps influenced by the difficulty of conducting EBNP upfront, current explorations of barriers and facilitators of EBNP among Chinese practitioners have focused primarily on how to conduct EBNP and have paid little attention to its sustainability after implementation. Our findings will generate potential factors to inform the design and implementation of sustainable EBNP.

First, during the sustainment phase, benefits to patients and professionals served as facilitators, encouraging nurse staff to engage in EBNP sustainability. For healthcare professionals, concern for the true impact and benefits to patients was a common motivator for dedicating to sustainability efforts. The findings are consistent with qualitative care studies that identify that Netherlandish nurse practitioners and doctors valued client‐centeredness and mentioned patients’ needs and satisfaction, thereby determining the sustainability of programs [31]. Furthermore, interviewees were concerned about whether routinely practicing EBNP could benefit nurses by improving work efficiency. It aligns with literature that explores continued benefits, especially advantages for nurses as an essential factor of program sustainability [32]. Therefore, EBNP practitioners should communicate and visualize the benefits of EBNP to patients so that nurses can understand these advantages, thereby contributing to nurses’ competence and satisfaction. Moreover, to promote EBNP routinization and its long‐term sustainability in the clinical setting, practitioners should strengthen the process design and tools of EBNP when planning EBNP to avoid increasing nurses’ work pressure and to achieve improved work efficiency [33].

In efforts to promote EBNP, professionals presented low motivation of nurses to participate in EBNP, low compliance to implement EBNP, and even disapproval by doctors and nurses. Work issues such as staff shortages, heavy workloads, and staff turnover were identified as barriers for some participants in sustaining the EBNP. Zurynski et al. [13] and Cowie et al. [12] reviewed that staff resourcing, such as staff shortages and high staff turnover feature strongly as barriers to the sustainability of healthcare interventions and programs. If the staff level is low or the work burden increases, nurses do not have sufficient time and space to engage in extra EBNP in daily practice, which also leads to low compliance and low motivation to implement and sustain for nurses. During the interview, many nurses indicated that they did not receive any material or nonmaterial compensation for their EBNP or maintenance work. To enable nurses to engage in EBNP sustainability, healthcare institutes or managers should reward employees for completing EBNP through incentives, such as bonuses, commendations, and title promotions, which can increase nurses’ compliance and encourage them to actively participate in EBNP [34]. Increasing related bonuses or including EBP projects in the assessment and employment content might be a way to integrate project sustainability efforts within the professional duty.

Factors that promote long‐term sustainability from respondents’ views were training, incentives, and embedding information systems. This finding is consistent with previous reviews of barriers and facilitators to EBP in low‐ and middle‐income countries [7]. Fundamentally, our study showed that organizational commitment over time would foster a supportive implementation climate for long‐term sustainability through accountability structures, incentives, and resource support [35, 36]. Interviews identified that disagreement with participation in maintenance or low adherence could also be attributed to a lack of relevant knowledge. Similarly, Iira et al. [37] identified that Finnish nurses do not have sufficient knowledge and training to provide evidence‐based care and to ensure sustainability in care and education. Training enables nurses to acquire the knowledge and skills needed to implement the EBNP and improves nurses’ self‐efficacy [15, 38, 39]. This suggests that training with a continuous planning perspective is needed to ensure adequate training and constant basic nursing education for both practicing nurses and new nurses and maintain fidelity [40]. Moreover, embedding the EBNP process into the information system not only reduces nurses’ work pressure but also provides reminders to urge nurses to complete the EBNP [41]. At the same time, organizations should support and assist innovation deliverers in improving their digital competence and enhancing their positive experience of using digital technologies [42].

Nurses identified that key supports of leaders, including the hospital’s senior leadership and head nurses, enable their ability to advance the sustainability of EBNP. These views are reflected in establishing the significant role of gaining multilevel leadership support in the long‐term sustainability of EBNP. Fleiszer et al. [32] and Luo et al. [43] found that leadership is a precondition for sustainability when formal leaders agree to EBNP and delegate delivery to middle managers [44]. Gould et al. [45] identified that supportive leadership was the most frequently reported facilitator for embedding the intervention into daily routine practice. Melnyk and Gallagher‐Ford [46] also note that some studies indicate that nursing managers may be facilitators of EBP, while others suggest they may be barriers. This may be reinforced in Chinese culture by the vertical clinical nursing power structure, in which the head nurse has significant power over the department and its nurses, and can decide on the implementation of the EBNP. Not only the support of leads but also the leadership of leaders, including evidence‐based knowledge, managerial skills, and a perception of change, is another facilitator of sustaining EBNP. Leadership lacking skills and concepts may negatively impact project sustainability by viewing it as a short‐term task or focusing solely on research results. It is similar to the recent study [47]. To address the constraint of leadership, it is recommended to conduct some online and offline training opportunities about how to strengthen leadership.

Through a mixed‐methods approach, we gained a better understanding of factors influencing the sustainability of the EBNP over varying periods. Our findings demonstrated that some barriers and facilitators are rarely mentioned when EBNP has been applied to their daily clinical routine for more than 3 years, whereas other factors are mentioned repeatedly at multiple stages. This situation may be addressed through interactions among factors that can facilitate sustainability while overcoming barriers to sustainability. For example, if there are facilitators like enhanced training, lack of knowledge among nurses is no longer a barrier to sustainability of EBNP. Previous literature in both hospitals and acute settings has highlighted that influencers on sustainability need to be considered dynamically and reciprocally [3, 48]. Therefore, we advocate continuous dynamic assessment of the factors affecting the EBNP and a greater understanding of the interactions among the determinants of sustainability.

### 4.1. Strengths and Limitations of the Work

This is a mixed‐methods study using both interviews and surveys to evaluate barriers and facilitators influencing sustainability. Both the answers and comments to open‐ended questions and qualitative data are used to further clarify and explain the relevant factors, which can be more comprehensive. Since we investigated the nurses from different regions of China and different grades of hospitals, we focused on changes in barriers and facilitators to EBNP at different stages, as well as barriers to sustainability that are prevalent in evidence‐based care practice. Caution is needed to interpret the results of this study. First, although we used CFIR to extract and categorize the determinants to make the barriers and facilitators more generalizable, the findings are still significantly influenced by the EBNP project itself and the implementation scenario. Second, the survey sample was not random, which may have led some eligible EBNP practitioners to not participate in the study. Participants came from 19 Chinese cities; the majority worked in tertiary hospitals, which may limit its generalizability to primary healthcare settings. Therefore, the universality of the research findings is limited. Third, while data saturation was reached, some interviews were relatively short (mean duration 30 min and range: 22–48 min), and the depth of interview questions varied by participants’ roles and experiences. As a result, certain CFIR questions may have been underexplored. This may potentially limit the richness and nuance of the data. This limitation was partially mitigated by the mixed‐methods design and by triangulating findings with open‐ended survey responses. Fourth, the time gap between the quantitative survey and the qualitative interviews is a limitation. As determinants of EBNP implementation may shift over time, the interview findings may reflect later‐stage perspectives rather than the earlier context captured in the survey. Fifth, while the translation process was meticulous, subtle linguistic and cultural nuances may not have been fully captured in English, potentially influencing the interpretation of qualitative data. Thus, further investigation is needed about theories or frameworks specifically for factors influencing sustainability of EBNP among nurses and combining them with actions and strategies to advocate sustainable EBNP.

## 5. Conclusion

This study explored the determinants of EBNP sustainability from the perspective of EBNP practitioners. The main barriers involved the inner setting and individual domain, including insufficient nurses, heavy workload, lack of leader support, cost, and limited communication with other multidisciplinary teams. For nursing practice, this highlights the urgent need for leadership, embedding the EBNP into the electronic system to simplify EBNP, adapting to the daily routines of the medical staff, enhancing regular training, and providing matching incentives are essential to EBNP sustainability. At the policy level, leaders and administrators should identify and dynamically explore related barriers and provide lessons for designing strategies and programs that promote the sustainability of EBNP in China. Quality control measures are needed to maintain fidelity and sustainability. To further strengthen EBNP sustainability in China, future research should focus on conducting longitudinal studies to explore how barriers and facilitators evolve across different phases of sustainment. Intervention studies testing targeted strategies to overcome identified barriers are also necessary. Additionally, the development of China‐specific sustainability assessment tools would be valuable.

## Author Contributions

Yanni Wu and Jingxia Miao: conceptualization, methodology, supervision, writing–original draft, and writing–review and editing. Jie Lai, Yujie Zhang, Jing Chi, Jiaqi Fu, Shisi Deng, Zihan Guo, Chuhan Zhong, Jianyao Tang, and Tingting Yang: formal analysis and investigation. Jie Lai, Yujie Zhang, Jing Chi, Hua Lin, Weixiang Luo, and Yanni Wu: writing–original draft and writing–review and editing. All authors commented on subsequent versions of the manuscript. Jie Lai, Yujie Zhang, Jing Chi, Jingxia Miao, Hua Lin, and Weixiang Luo contributed equally to this work.

## Funding

This project was funded by the National Natural Science Foundation of China (nos. 72304131 and 72574095), Guangdong Basic and Applied Basic Research Foundation (2025A1515012965), and the Outstanding Youths Development Scheme of Nanfang Hospital, Southern Medical University (no. 2023J005).

## Disclosure

The funder had no role in study design, data collection and analysis, the decision to publish, or preparation of the manuscript. All authors have approved the final manuscript.

## Ethics Statement

Ethical approval for this study was obtained from the Medical Ethics Committee of Nanfang Hospital, Southern Medical University, Guangzhou, China (Ethical number: NFEC‐2021‐439).

## Conflicts of Interest

The authors declare no conflicts of interest.

## Supporting Information

Additional supporting information can be found online in the Supporting Information section.

## Supporting information


**Supporting Information 1** Supporting file 1: Consolidated Criteria for Reporting Qualitative Research (COREQ) checklist. Description of Supporting file 1: Supporting file 1 presents a checklist according to the COREQ guidelines that confirms that qualitative part meets the appropriate qualitative research report.

**Supporting Information 2** Supporting file 2: Good reporting of a mixed‐methods study (GRAMMS) checklist. Description of Supporting file 2: Supporting file 2 presents a checklist to the GRAMMS that confirms the quality of mixed methods studies in health services research.

**Supporting Information 3** Supporting file 3: Interview guide. Description of Supporting file 3: Supporting file 3 describes an interview guide created based on the five domains of the Consolidated Framework for Implementation Research (CFIR) that supported semistructured interviews.

**Supporting Information 4** Supporting file 4: Mixed‐method joint display integration. Description of Supporting file 4: Supporting file 4 presents a mixed‐method joint display depicting barriers and facilitators on sustainability of EBNP.

## Data Availability

The data that support the findings of this study are available from the corresponding author upon reasonable request.
