# Supplementary material for: Perceptions of Factors Associated With Sustainability of Evidence‐Based Nursing Practice: A Sequential Mixed Methods Study
Source: J Nurs Manag. 2026 May 15;2026:6680206. doi: 10.1155/jonm/6680206 (PMC13176852; doi:10.1155/jonm/6680206)
Supplement: Supplementary file 2 — Supporting Information 2 Supporting file 2: Good reporting of a mixed‐methods study (GRAMMS) checklist. Description of Supporting file 2: Supporting file 2 presents a checklist to the GRAMMS that confirms the quality of mixed methods studies in health services research. [file JONM-2026-6680206-s002.pdf]

Supporting file 2: Good reporting of a mixed-methods study (GRAMMS) checklist

A checklist to the GRAMMS (Good reporting of a mixed-methods study) that confirm that quality of mixed methods studies in health services research

Guideline	Page information
1. Describe the justification for using a mixed methods approach to the research question	4
2. Describe the design in terms of the purpose, priority and sequence of methods	4-5
3. Describe each method in terms of sampling, data collection and analysis	5-7
4. Describe where integration has occurred, how it has occurred and who has participated in it	8
5. Describe any limitation of one method associated with the present of the other method	18
6. Describe any insights gained from mixing or integrating methods	9-15

Reference: O'Cathain A, Murphy E, Nicholl J. The quality of mixed methods studies in health services research. J Health Serv Res Policy. 2008;13: 92-98.
