# Supplementary material for: Perceptions of Factors Associated With Sustainability of Evidence‐Based Nursing Practice: A Sequential Mixed Methods Study
Source: J Nurs Manag. 2026 May 15;2026:6680206. doi: 10.1155/jonm/6680206 (PMC13176852; doi:10.1155/jonm/6680206)
Supplement: Supplementary file 3 — Supporting Information 3 Supporting file 3: Interview guide. Description of Supporting file 3: Supporting file 3 describes an interview guide created based on the five domains of the Consolidated Framework for Implementation Research (CFIR) that supported semistructured interviews. [file JONM-2026-6680206-s003.docx]

**Supporting file 3**

A interview guide created based on the five domains of consolidated framework for implementation research (CFIR) framework that supported semi-structured interview

**Interview guide**

1. How has EBP changed your usual daily practices? What are the main reasons for these changes?
2. What changes are needed to sustain EBP or integrate it into daily practice?
3. In your opinion, how does the complexity of the EBP project impact its sustainability or integration into daily practice?
4. What supports, such as online resources, marketing materials, or toolkits, are available to help you sustain the EBP project or integrate it into daily practice?
5. What costs do you think are needed to sustain EBP?
6. To what extent do you consider the needs and preferences of patients when deciding to sustain EBP or integrate it into daily practice?
7. How does your network with external organizations affect the sustainability of EBP?
8. What is your opinion on any resources, support, or policies that could affect the sustainability or integration of EBP into daily practice?
9. How does the infrastructure of your organization (e.g., social architecture, age, maturity, size, or physical layout) affect the sustainability of the intervention?
10. To what extent do you think integrating EBP into routine practice can meet the needs of the organization?
11. Can you describe the relationship between you and your colleagues in your department? How does it affect the sustainability of EBP?
12. What kind of incentives or motivations are needed to sustain the EBP project?
13. What role does internal supervision play? To what extent do you receive support from that supervision? What resources do you need to sustain the EBP project?
14. What challenges do you expect to encounter?
15. How do you and your colleagues feel about the intervention being used in your setting?
16. How confident are you and your colleagues that you can successfully sustain this EBP or integrate it into daily practice?
17. What is the attitude of you and your team members toward sustaining the intervention and integrating it into daily practice?
18. What plans have you made to integrate EBP into routine practice?
19. What are the greatest barriers and facilitators you have encountered in sustaining EBP or integrating it into daily practice?
20. What are your recommendations regarding strategies to facilitate the sustainability of EBP?
