# Supplementary material for: Perceptions of Factors Associated With Sustainability of Evidence‐Based Nursing Practice: A Sequential Mixed Methods Study
Source: J Nurs Manag. 2026 May 15;2026:6680206. doi: 10.1155/jonm/6680206 (PMC13176852; doi:10.1155/jonm/6680206)
Supplement: Supplementary file 4 — Supporting Information 4 Supporting file 4: Mixed‐method joint display integration. Description of Supporting file 4: Supporting file 4 presents a mixed‐method joint display depicting barriers and facilitators on sustainability of EBNP. [file JONM-2026-6680206-s004.docx]

Supporting file 4. Mixed-method joint display integration

|  | Construct | Quantitative findings | Supporting illustrative quotes | Interpretation |
| --- | --- | --- | --- | --- |
| **Innovation** | | | | |
|  | Innovation cost | Increased work costs (28/394, 7.11%) were identified as a barrier and reported across 6 of 7 stages of evidence implementation. | “*Standardized tools increase costs and expenses, and spending is not things we can control.” (P2)*  “*But more and more patients can …receive professional management…, which generates greater social value*” *(P9)* | Innovation cost may be a barrier when additional expenditures are not well supported, but participants weighed the immediate financial expense against potential social value. |
|  | Innovation relative advantage | Benefits to patients (24/377, 6.37%) and improved work efficiency (17/377, 4.51%) were identified as facilitators.  These were reported repeatedly across more than 5 of the 7 stages of evidence implementation. | *“The most important point is the embodiment of the effect. After our efforts, the qualified rate is improved” (P6)* | EBNP is more likely to be sustained when nurses perceive clear relative advantages |
| **Inner setting** | | | | |
|  | Work Infrastructure | Shortage of nurse staffing (41/394, 10.41%) and heavy work pressure (55/394, 13.96%) are common barriers.  Embedded in information system was reported as a facilitator across 6 of 7 implementation stages. | *“The hospital is understaffed, and nurses are already overburdened. Implementing this strategy will increase their workload.” (P11)*  *“Integrated into electronic information systems, it has become a routine used tool” (P4)* | Inadequate staffing hindered sustainability, while HIS integration improved workflow fit and adherence. |
|  | Relational Connections | Departmental teamwork (27/377, 7.16%) was identified as a facilitator, whereas lack of team cooperation (28/394, 7.11%) was identified as a barrier. | *“One department has done an excellent job with EBP. When sharing their experiences, they mentioned that the collaboration between doctors and nurses is outstanding.” (P1)*  *“Everyone has different roles, different responsibilities, and different priorities, conflicting viewpoints, and communication barriers.” (P11)* | EBNP sustainability depended on effective multidisciplinary collaboration; supportive teamwork was facilitated, whereas poor communication and role conflicts hindered long-term implementation. |
|  | Incentive systems | Incentives (8/377, 3.12 %) were identified as a facilitator and were reported across 5 of the 7 stages of evidence implementation. | *“If you succeed in completing it, you will get an extra point, and if you fail to complete it, one point will be deducted.” (P15)* | Structured incentive systems can strengthen EBNP sustainability by linking participation and performance with rewards, recognition, and accountability. |
|  | Access to knowledge & information | Enhanced training (12/377, 3.18%) was identified as a facilitator and was reported across 5 of the 7 stages of evidence implementation. | *“If we provide doctors and nurses with the latest information, they will appreciate it and be eager to learn new things.” (P10)*  *“We plan to provide orientation to new nurses when they join the department.” (P12)* | Diversified training can embed EBNP into routine professional development and strengthen staff competence. |
|  | Leadership and governance commitment | N/A | *“The hospital believes consistency is key; if direct supervisors are committed to doing their jobs well, they will succeed.” (P2)*  *“It is actually quite difficult to get the hospital to endorse a nursing reform project; it is just a passing fad” (P1)* | Leadership and governance commitment influenced whether EBNP was treated as a long-term priority, thereby shaping staff engagement, adherence, and sustainability. |
|  | High-level and mid-level leaders | Supported by leadership (92/377, 24.4%) was a facilitator, while lack of leadership support (33/394, 8.38%) was reported as a barrier.  Leadership of leaders (19/377, 5.04%) can be a facilitator. | *“Since the head nurse didn't t support me, I’ ll continue to seek support from higher-level management. I’ ll ask the director of nursing to back me up” (P12)*  *“I didn't express my leadership. I relied too much on my director to issue orders and announcements.” (P10)* | EBNP sustainability depended not only on leaders’ endorsement and resource support but also on their active leadership in mobilizing staff, coordinating implementation, and sustaining a change-supportive climate. |
|  | Capability and motivation | Capability- and motivation-related barriers were repeatedly reported across more than five of the seven implementation stages, including lack of EBNP knowledge among nurses (11/394, 2.79%), disapproval of EBNP by doctors and nurses (19/394, 4.82%), low compliance (17/394, 4.31%), and low enthusiasm (13/394, 3.30%). | *“Due to the influence of academic background and seniority, some nurses are mainly empiric, do not recognize the significance of evidence-based practice” (P11)*  *“Remind the doctor to adjust their approach; the doctor doesn't seem very happy.” (P3)* | Nurses identified that EBNP sustainability depended on whether staff had both the capability to understand and accept EBNP and the motivation to continue implementing it in practice. |
|  |  |  |  |  |
|  | Opinion leaders and implementation leaders | Three nurses (0.8%) reported that project implementation leaders' persistence was a facilitator.  Only one nurse (0.27%) reported that nurses have the authority to lead as a facilitator. | *“If my title again promotion, a little more working years, a little more senior may be a little bit more persuasive.” (P11)*  *“In a group setting, when an opinion leader emerges, others tend to follow suit, it can be either positive or negative.” (P4)* | EBNP sustainability depends not only on formally appointed implementation leaders, but also on the informal influence of opinion leaders. |
| **Process** | | | | |
|  | Tailoring strategies | Only one nurse (0.27%) from a project lasting less than a year identified continuity of implementation strategy as a facilitator. | *“It is still necessary to gradually adjust and refine strategies according to your clinical practice.”(P5)* | Although less frequently reported in the survey, continuous strategy adjustment is important for sustaining EBNP, and evaluation needs evolve across implementation stages. |
|  | Assessing needs | N/A | *“If you don't dig deep into doctors' and nurses’ ideas and simply use our daily informal communication… Because deep factors are not expressed.” (P3)* | The needs assessment was crucial for understanding hidden barriers and informing strategic intervention points. |
|  | Reflecting & evaluating | Quality control measures were reported as a facilitator (12/377, 3.18%), while 10/394 (2.53%) were identified as barriers. | “*At least four rounds of follow-up supervision to maintain and solidify the best practice” (P4)*  “*A ‘take it one step at a time’ mindset doesn't really take sustainability into account.” (N11)* | The nurses noted that reflecting and evaluating are beneficial when structured and sustained, but they become barriers when sustainability is not intentionally built into planning and monitoring processes. |
| **Outer setting** | | | | |
|  | Policies and laws | Policy support (9/377, 2.39%) was identified as a facilitator. | *“…national policies such as healthy China, they are moving forward” (P3)*  *“Hospitals and industries all conduct annual evaluations, carried out as routine, making it easier to pass” (P10)* | Survey respondents and the qualitative data suggest that national policy initiatives and external evaluation mechanisms could create a favorable environment for sustaining EBNP |
|  | Partnerships and connections | Organizational support (10/377, 2.65%) was reported as a facilitator. | *“established the evidence application base in collaboration with the university” (P14)*  *“Organize activities with the association to play a leading role” (P10)* | Organizational support from academic organizations can provide expertise, visibility, and opportunities for the sustainable development of EBNP. |

Note: N/A: Not captured as a distinct quantitative item.
